# Establishment and evaluation of a stable CHO cell line in which the nanobody PD-L1-Fc gene is precisely targeted into the C12orf35 locus: Establishment and evaluation of a targeted integrated CHO cell line

**DOI:** 10.3724/abbs.2025187

**Published:** 2025-10-10

**Authors:** Feng Chang, Chen Zhang, Yu Feng, Wenyun Zheng, Xingyuan Ma

**Affiliations:** 1 State Key Laboratory of Bioreactor Engineering School of Biotechnology East China University of Science and Technology Shanghai 200237 China; 2 Shanghai Key Laboratory of New Drug Design School of Pharmacy East China University of Science and Technology Shanghai 200237 China

Chinese hamster ovary (CHO) cells are an extensively used platform for manufacturing biopharmaceuticals, and nearly 80% of recombinant protein is produced by CHO cell lines [1]. Randomly incorporating genes of interest into the genome is a common method for the development of stable CHO cell lines in industry, but it is vulnerable to genetic instability, is difficult to predict productivity, and is accompanied by a time-consuming and laborious screening process, [2]. Nonetheless, highly productive clones isolated from a randomized pool often exhibit unfavorable properties including transgene copy number loss and epigenetic silencing over the lifespan of the culture, ultimately lowering transgene transcription and corresponding recombinant protein production, which referred to as production instability [ 3, 4] . Thus, this challenging situation underscores the urgent desire for a new strategy to satisfy ever-growing industrial production requirements.

The lack of specificity of gene integration, which is often susceptible to genetic instability, causes production instability. Alternatively, in recent years, many investigators have shown that the bottleneck arising from traditional randomized cell line development can be overcome through site-specific integration to insert exogenous pieces of DNA into a precise location, which permits their predictable function, allows high levels of transgene expression and makes it possible to generate homogeneous clones with consistent productivity and stability [ 5, 6] . The transcription and expression activities of genes are influenced by chromatin structural properties and the environment surrounding the genome, and loci that are capable of facilitating high and stable transgene transcription and expression are termed “hotspots” [7]. Many significant upfront advances have been made to identify potential hotspots, and a number of promising genome loci have been reported, such as the Hprt, Ywhae, Hipp11, Rosa26, and C12orf35 loci [8]. The C12orf35 gene is located on a telomeric region of chromosome 8 in CHO cells. It is widely known that telomeres are usually noncoding, repetitive sequences distributed at chromosome terminals that act as buffers for those coding sequences further behind and thus enable foreign gene expression without interrupting functional genes. Studies have demonstrated that the C12orf35 gene is a potential locus for the integration of foreign genes in mammalian cells and that disruption of C12orf35 gene expression leads to increased productivities and shorter recovery times during selection pressure in CHO cells [ 9, 10] . Although the C12orf35 gene has been partially researched in cell line development, very few publicly available reports have systematically validated site-specific integration in cell lines concerning the stability of transgene passage, transgene transcription and expression levels.

A range of studies have successfully utilized site-specific recombinase or genome editing tools to incorporate exogenous genes into the desired site in the CHO genome. Clustered regularly interspaced short palindromic repeats/Cas9 (CRISPR-Cas9), a leading gene editing tool, uses a guide RNA to target the DNA sequence with the Cas9 enzyme to induce cuts and allows easy, efficient and cost-effective edting. CRISPR-Cas9 has already been applied to mediate the insertion of targeted genes in mammalian cells, including CHO cells, for fundamental research [5]. However, the adoption of this technology for industrial purposes remains to be investigated. Therefore, in this study, we sought to establish a CRISPR/Cas9-mediated site-specific integration strategy to overcome existing weaknesses and lay the foundation for the development of industrial rCHO cell lines.

**Figure 1:**
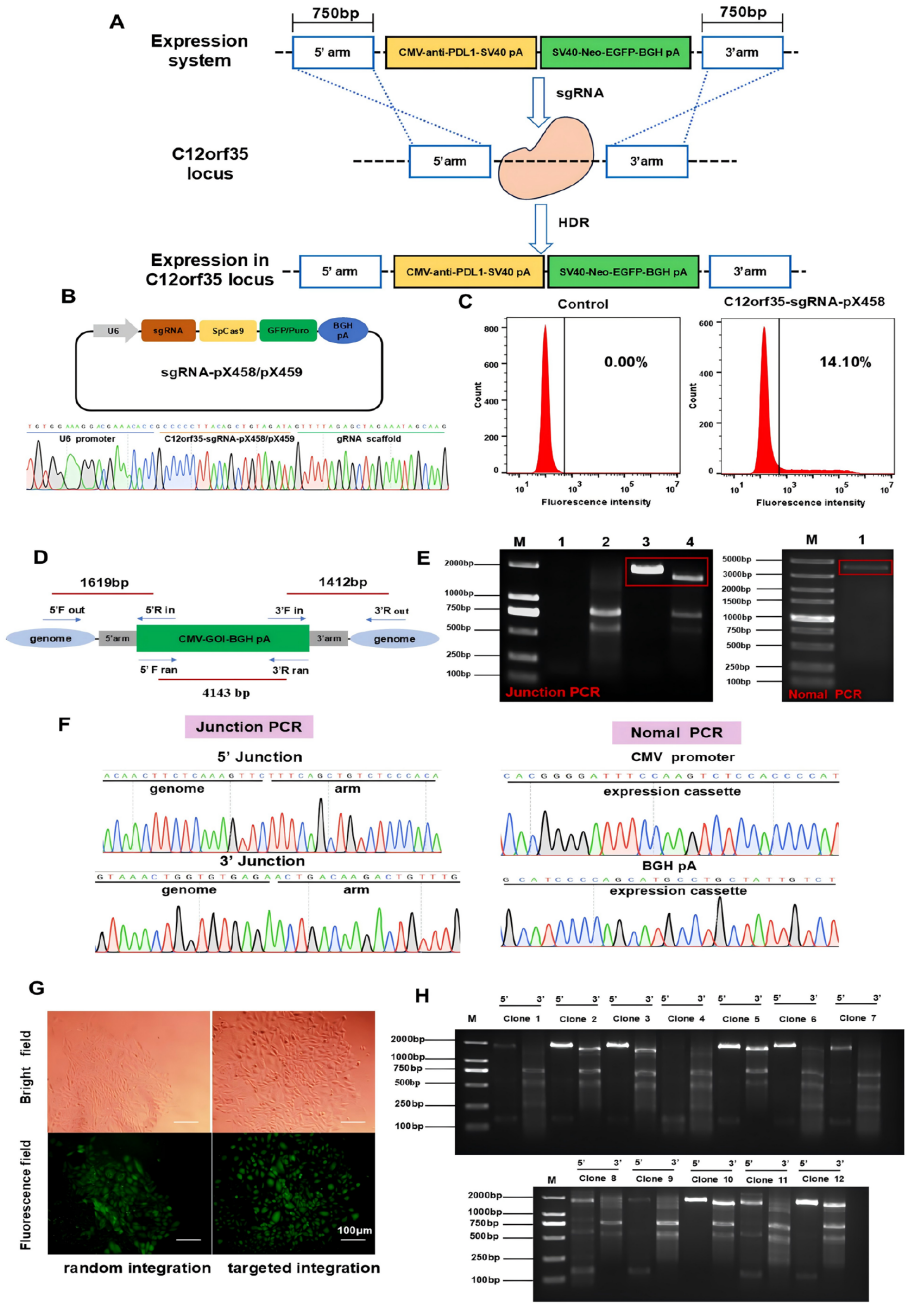
Figure 1 Establishment of the stable CHO cell line of targeted integration (A) Schematic diagram of CRISPR/Cas9-mediated targeted integration of expression cassette. (B) The construction for the sgRNA-pX458/pX459 tool vector. (C) Transfection efficiency of C12orf35-sgRNA-pX458 plasmid. (D) Schematic diagram of cell lines with targeted and randomized integration identified by 5′/3′ Junction PCR and normal PCR. (E) Electrophoretic image of 5′/3′ Junction PCR and normal PCR for targeted and randomized cell pool. Lane M: DL2000 Marker; Lane 1: 5′ Junction PCR for random integration group; Lane 2: 3′ Junction PCR for random integration group; Lane 3: 5′ Junction PCR for targeted integration group; Lane 4: 3′ Junction PCR for targeted integration group. Lane M: DL5000 Marker; Lane 1: Normal PCR for random integration group. (F) Sequencing diagram of target bands for 5′/3′ Junction PCR in targeted integration cell pool and normal PCR in targeted integration cell pool. (G) Expression of EGFP after screening for 3 weeks. (H) 5′/3′ Junction PCR identification of targeted integration for monoclonal cell lines.

We constructed a homologous expression vector comprising the CMV promoter, anti-PDL1 (Generay, Shanghai, China), SV40 pA, SV40 promoter, EGFP, Neo and BGH pA. There is a 750 bp homology arm upstream of the CMV promoter and downstream of the BGH pA ( Figure 1A). Importantly, EGFP is capable of monitoring the expression of exogenous genes in real time and visualizing the process of screening cell lines. The oligonucleotide sgRNAs (Tsingke, Beijing,China) were synthesized, annealed and ligated into the pX458 plasmid (Addgene, Watertown, USA) ( Figure 1B). After 48 h of transfection of the pX458-sgRNA plasmid into CHO-K1 cells, we performed flow cytometry analysis and observed a transfection efficiency of 14.1%, which could guarantee the occurrence of genome cleavage ( Figure 1C). Thus, we constructed a pX459-sgRNA plasmid and used its targeted cleavage position as an exogenous gene insertion site.

The pX459-sgRNA plasmid and linear expression cassette were co-transfected into CHO-K1 cells, and an equivalent number of donor plasmids were used for single transfection. Approximately, 48 h post-transfection, the medium containing G418 was replaced, and a pool of Neo-resistant cells was obtained via the G418 screening process for 3 weeks. Afterwards, the genomic DNA was extracted from the stably transfected cell pool via the Genome Extraction Kit (Tiangen, Beijing, China) and subjected to 5′/3′ junction PCR with forward and reverse primers designed to be on the outside of the 5′/3′ homology arm and inside the expression cassette, respectively. In addition, normal PCR for the genomic DNA of the randomized integrated cell pool was performed with a 5′ F ran forward primer matching the CMV promoter and a 3′ R ran reverse primer matching the BGH pA ( Figure 1D). The PCR image suggested that the targeted gene fragment of the site-specific pool was successfully obtained, and the 4143 bp desired gene fragment from the randomized pool of DNA was also favorably amplified ( Figure 1E). The sequencing analysis of the amplified gene fragment further confirmed the successful insertion of the exogenous gene into the C12orf35 locus, and non-homologous random integration occurred in the randomized cell pool ( Figure 1F). Owing to the expression cassette containing EGFP, the enrichment of the positive cell population was also monitored via inverted fluorescence microscopy ( Figure 1G). Then, the cell pools were seeded at 1 cell/well in a 96-well plate by limiting dilution. After another 3‒4 weeks, 6 monoclonal cell strains with random interaction, namely, CHO-RA-1, CHO-RA-2, CHO-RA-3, CHO-RA-4, CHO-RA-5 and CHO-RA-6, were obtained. For targeted integration, the CHO-SA-2, CHO-SA-3 CHO-SA-5, CHO-SA-10 and CHO-SA-12 clones were further verified via 5′/3′ junction PCR and DNA sequencing, and 6 out of the 12 clones were successfully inserted ( Figure 1H). These results demonstrated that the C12orf35 locus could be used for site-specific integration of exogenous genes with a targeting efficiency of 42%.

**Figure 2:**
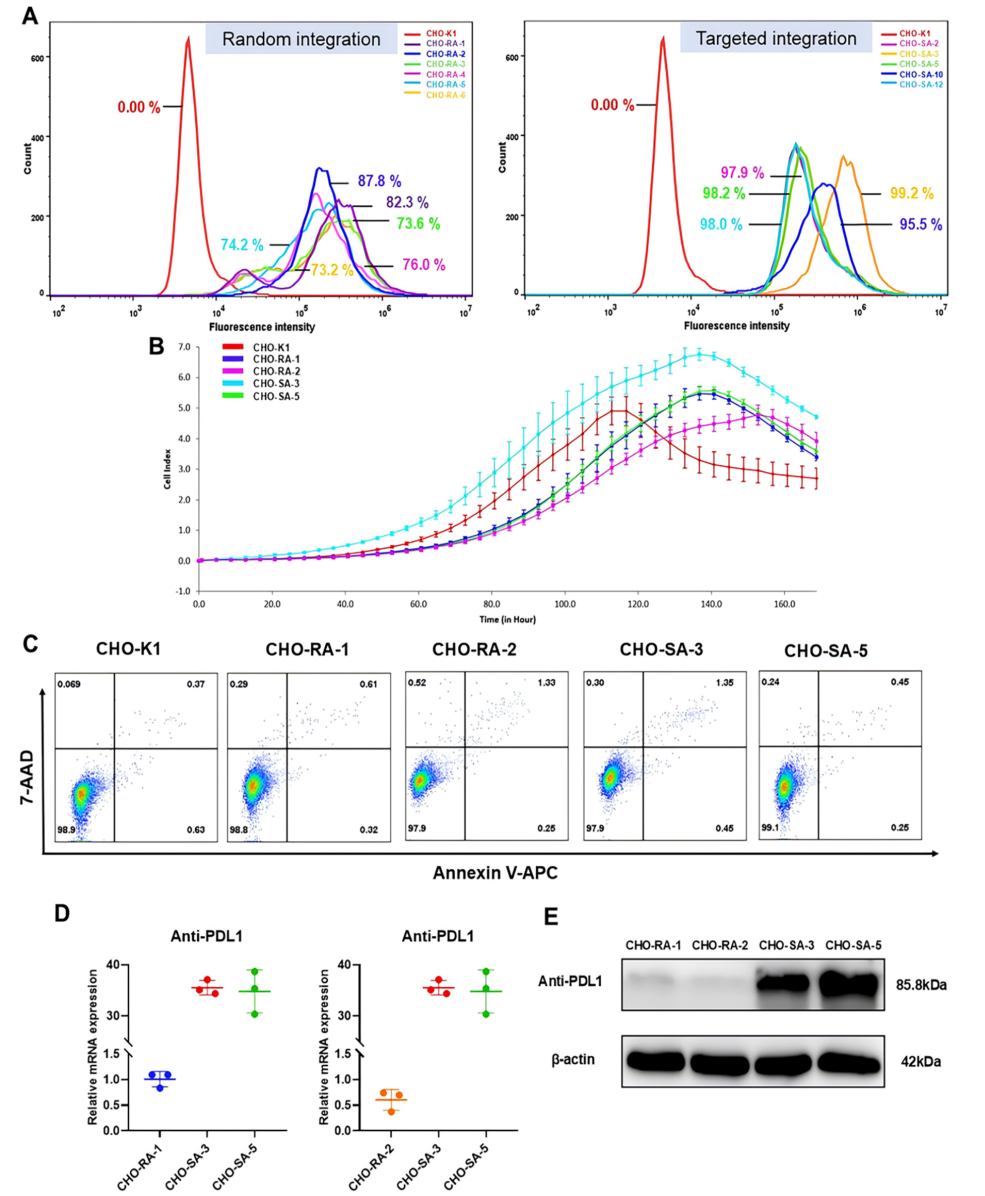
Figure 2 Evaluation of the stable CHO cell line of targeted integration (A) Stability of positive monoclonal cell lines for passage. (B) Proliferation of targeted integration cell lines detected by RTCA. Data are expressed as the mean ± SD (n = 3). (C) The apoptosis rate was analyzed by FCM. (D) Relative mRNA expression level of anti-PDL gene in CHO-RA-1, CHO-RA-2, CHO-SA-3, and CHO-SA-5 cell lines detected by qPCR. Data are expressed as the mean ± SD (n = 3). (E) Anti-PDL1 protein expression level in CHO-RA-1, CHO-RA-2, CHO-SA-3, and CHO-SA-5 cell lines detected by western blot analysis.

To determine the gene expression stability of the cell lines, we detected the proportion of EGFP-positive cells in the entire population at the 20th passage via flow cytometry and found that different degrees of exogenous gene expression silencing occurred in the randomized group. After culture for 20 passages, the highest percentage of EGFP-positive cells in the random group was only 87.8%, whereas the minimum percentage in the site-specific group was 95.5% ( Figure 2A). These data demonstrated that, compared with traditional approaches, the targeting of exogenous genes into the C12orf35 locus overcomes the considerable obstacle in terms of genetic expression stability [ 3, 4] . Since the cell lines presented differences in expression stability, we selected four more stable cell lines, CHO-RA-1 and CHO-RA-2, from the randomized group and CHO-SA-3 and CHO-SA-5, from the site-specific group for subsequent experiments. To evaluate whether C12orf35 is a safe locus, a cell proliferation assay was performed with an xCELLigence RTCA instrument (Agilent, Santa Clara, USA). A total of 1×10 ^3^ cells were seeded in an E-Plate 16 and cultured continuously for 7 days. Each sample was set up in triplicate and recorded at 4 h intervals. As depicted in Figure 2B, compared with wild-type CHO-K1 or randomized clones, the targeted clones did not show obvious growth retardation or arrest. In addition, although the exogenous gene was inserted into the same position in the C12orf35 locus, the growth rate and tendency of the two site-specific clones were not greater than those of the wild-type CHO-K1 or randomized clones. To further confirm the safety of the C12orf35 site, we also conducted an apoptosis test via flow cytometry. The cells were harvested and resuspended in 100 μL of 1× Annexin V binding buffer. The cells were subsequently supplemented with 2.5 μL of Annexin V-APC and 2.5 μL of 7-AAD reagents (Elabscience, Wuhan, China) and incubated for 15 min at room temperature in the dark. A total of 400 μL of 1× Annexin V binding buffer was added, gently and evenly pipetted, and detected by a machine. After analysis, we found that the apoptosis rate was not significantly different between site-specific clones and randomized clones or wild-type CHO-K1 cells ( Figure 2C). These results confirmed that the C12orf35 locus is not involved in the regulation of cellular growth and is a safe site for integration. To evaluate whether the C12orf35 locus is an active transcription region, the relative mRNA levels of the anti-PDL1 gene in the CHO-RA-1, CHO-RA-2, CHO-SA-3 and CHO-SA-5 cell strains were detected. In this experiment, the 20-μL qPCR system contained 4 μL of DNA template, 0.4 μL of forward and reverse primers, and 10 μL of SYBR Green qPCR Master Mix (Vazyme, Nanjing, China). The amplification conditions were as follows: 95°C for 30 s; 40 × 95 °C for 10 s; and 60°C for 30 s. For the anti-PDL1 mRNA expression level, two targeted integrated clones presented 30-fold higher expression than CHO-RA-1 and 50-fold higher expression than CHO-RA-2 did ( Figure 2D), which revealed that the C12orf35 locus is an active transcription region. The higher transcription level of the exogenous gene means that there may be a higher protein expression level, which is very beneficial for r-protein production. After that, we further confirmed whether the C12orf35 locus was highly expressed. A comparison was conducted between the protein expression of the targeted and randomized integrated clones via western blot analysis. Target cells were lysed with Cell Lysis Buffer (Beyotime, Shanghai, China) containing 1 mM PMSF on ice, the lysed supernatant was collected, and the total protein concentration was measured with a BCA protein assay kit (Biosharp, Beijing, China). The samples were boiled for 10 min, separated by 10% SDS‒PAGE, and then transferred to 0.45-μm PVDF membranes (Beyotime). After blocking with 5% skim milk at 4°C overnight, the membrane was incubated with an HRP-conjugated AffiniPure goat anti-human IgG (H+L) antibody (Proeintech, Wuhan, China) for 1 h at room temperature and visualized via an enhanced chemiluminescence (ECL) chemiluminescence kit (Sangon Biotech, Shanghai, China). As shown in Figure 2E, the anti-PDL1 expression level at the C12orf35 locus was significantly greater than that in the randomly integrated cell strains CHO-RA-1 and CHO-RA-2. We could conclude that, in terms of the protein expression level of the exogenous gene, the site-specific integration clones were more advantageous, which proved that the C12orf35 locus is a high protein expression region. This is another good way to increase productivity.

In summary, we successfully incorporated an exogenous gene into the C12orf35 locus via CRISPR/Cas9 genome-editing technology and evaluated the performance of site-specific integrated cell strains through a myriad of assays. Our study systematically confirmed that the C12orf35 locus is a stable gene expression region, active transcription region and high protein expression region and that the C12orf35 locus is not involved in the regulation of cell growth and is a safe integration site. These findings indicate that targeting exogenous genes into the C12orf35 locus is a feasible way to develop engineered cell lines for efficient production.
